# Supplementary material for: Maternal Acylcarnitine Disruption as a Potential Predictor of Preterm Birth in Primigravida: A Preliminary Investigation
Source: Nutrients. 2024 Feb 22;16(5):595. doi: 10.3390/nu16050595 (PMC10934651; doi:10.3390/nu16050595)
Supplement: Supplementary file 1 [file nutrients-16-00595-s001.zip › nutrients-2833062-supplementary.pdf]

Table S1: Annotated Metabolites

Metabolites with VIP>1	Fold Change[1]	*p*-value	FDR *q*-value at 15%	VIP Score
Dopamine	1.533	**0.0013**	**0.0905**	1.230
Butenylcarnitine (C4:1)	2.093	**0.0015**	**0.0905**	2.272
Tyrosine	1.447	**0.0015**	**0.0905**	1.200
Decadienoylcarnitine (C10:2)	1.482	**0.0023**	**0.1041**	1.141
Threonylthreonine	1.478	**0.0032**	**0.1072**	1.250
Dodec-enedioylcarnitine (C12:1-DC)	1.461	**0.0037**	**0.1072**	1.311
Glutaminyllysine	1.572	**0.0051**	**0.1072**	1.257
Undeca-trienoylcarnitine (C11:3)	1.510	**0.0055**	**0.1072**	1.564
Acetyl-L-arginine	1.386	**0.0061**	**0.1072**	1.231
Propenoylcarnitine (C3:1)	1.438	**0.0065**	**0.1072**	1.257
Octenoylcarnitine (C8:1)	1.328	**0.0067**	**0.1072**	1.045
Hept enoylcarnitine (C7:1)	1.479	**0.0074**	**0.1072**	1.439
Hydroxydecanoylcarnitine (C10:0-OH)	1.505	**0.0077**	**0.1072**	1.178
Hydroxyundecanoylcarnitine (C11:0-OH)	1.483	**0.0099**	**0.1207**	0.897
Methylhexanoylcarnitine (C6:0 M)	1.350	**0.0100**	**0.1207**	1.301
Alanine	1.465	**0.0108**	**0.1214**	1.546
Methyloctanoylcarnitine (C8:0 M)	1.448	**0.0114**	**0.1214**	1.443
Hydroxydodecanoylcarnitine (C12:0-OH)	1.408	**0.0151**	0.1518	0.921
Threonylphenylalanine	1.609	**0.0194**	0.1848	1.153
Hydroxyestradiol	-2.033	**0.0237**	0.2051	1.606
Phenylalanine	1.456	**0.0238**	0.2051	1.145
Undec-enoylcarnitine (C11:1)	1.411	**0.0268**	0.2205	0.819
Octadienoylglycine	1.408	**0.0287**	0.2240	0.844
Paraxanthine	2.463	**0.0297**	0.2240	2.147

**Metabolites with VIP>1**	**Fold Change[1]**	***p*-value**	**FDR *q*-value at 15%**	**VIP Score**
Lysylasparagine	1.312	**0.0354**	0.2534	1.295
Aspartyl-Valine	1.376	**0.0378**	0.2534	0.728
Methyldecanoylcarnitine (C10:0 M)	1.435	**0.0381**	0.2534	0.862
Glutamylserine	1.247	**0.0392**	0.2534	0.729
Non-enedioylcarnitine (C9:1-DC)	1.283	**0.0434**	0.2709	1.481
Epinephrine	1.426	0.0575	0.3469	0.761
Methylpentanoylcarnitine (C5:1 M)	1.439	0.0648	0.3783	1.285
Hydroxytrtradeca-dienoylcarnitine (C14:2-OH)	1.455	0.0677	0.3829	0.665
Hydroxypentanoylcarnitine (C5:0-OH)	1.344	0.0743	0.3939	0.852
Aspartylphenylalanine	1.772	0.0826	0.3939	1.066
Leucyl-Cysteine	1.241	0.0836	0.3939	1.325
Norepinephrine	1.233	0.0857	0.3939	0.966
Lysylmethionine	1.274	0.0873	0.3939	1.149
Nona-dienoylcarnitine (C9:2)	1.248	0.0877	0.3939	0.696
Oct-enedioylcarnitine (C8:1-DC)	1.245	0.0891	0.3939	0.808
Threonylasparagine	1.208	0.0895	0.3939	1.299
Asp-Gly-Lys	1.371	0.0913	0.3939	0.769
Glutaminylcysteine	1.344	0.0914	0.3939	1.150
Thymine	-1.132	0.0955	0.4020	1.338
L-Carnitine	-1.121	0.1000	0.4114	1.604
Tryptophyl-Proline	1.453	0.1074	0.4320	0.846
Kynurenic acid	1.446	0.1145	0.4479	0.927
Decenoylcarnitine (C10:1)	1.429	0.1212	0.4479	1.194
Valine	1.178	0.1220	0.4479	1.005

Metabolites with VIP>1	Fold Change[1]	*p*-value	FDR *q*-value at 15%	VIP Score
Androsterone	1.238	0.1224	0.4479	0.894
Phenylalanyltryptophan	1.380	0.1256	0.4479	0.788
Tetradecadienoylcarnitine (C14:2)	1.284	0.1262	0.4479	0.549
Histidylhistidine	1.345	0.1330	0.4629	0.658
Glutaminylglutamine	1.256	0.1373	0.4689	0.675
Biocytin	1.390	0.1493	0.4933	0.792
Xanthine	1.235	0.1499	0.4933	0.592
Prolylphenylalanine	1.245	0.1603	0.5181	1.358
Threonylproline	1.192	0.1660	0.5255	0.761
N-Acetylglutamine	1.207	0.1690	0.5255	0.612
Tyramine	1.400	0.1725	0.5255	0.638
Dodeca-dienoylcarnitine (C12:2)	1.301	0.1755	0.5255	1.497
Leucylleucine	1.335	0.1778	0.5255	0.667
Threonylalanine	1.298	0.1800	0.5255	0.973
Phenylalanylphenylalanine	1.307	0.1832	0.5263	0.514
5-Hydroxyoctanoylcarnitine (C10:0-OH)	1.529	0.1942	0.5492	1.313
Acetylcarnitine	1.437	0.2033	0.5661	0.737
Glutamylalanine	1.152	0.2097	0.5711	1.368
Hexenedioylcarnitine (C6:1-DC)	1.264	0.2114	0.5711	0.636
Piperidine	1.217	0.2173	0.5784	0.700
Hydroxyisovaleric acid	1.321	0.2392	0.6027	0.754
Glycyl-Aspartate	1.232	0.2412	0.6027	1.128
p-Cresol glucuronide	1.302	0.2479	0.6027	0.838
Glutamylphenylalanine	1.225	0.2486	0.6027	0.670
Leucine	1.248	0.2501	0.6027	0.743

Metabolites with VIP>1	Fold Change[1]	*p*-value	FDR *q*-value at 15%	VIP Score
Hydroxyhexanoycarnitine (C6:0-OH)	1.396	0.2516	0.6027	1.111
p-Cresol	1.162	0.2557	0.6027	0.647
Asparaginylglycine	1.303	0.2623	0.6027	0.910
Uric acid	-1.109	0.2664	0.6027	1.464
Octenoylglycine	1.229	0.2686	0.6027	0.604
DL-Dopa	1.106	0.2694	0.6027	0.860
Nonanedioylcarnitine (C9:0-DC)	1.254	0.2706	0.6027	0.787
Heptenoylcarnitine (C7:1)	1.515	0.2752	0.6027	1.611
Leucylphenylalanine	1.333	0.2792	0.6027	0.484
5-Aminopentanamide	1.212	0.2797	0.6027	0.702
alpha-Ketoisovaleric acid	1.145	0.2797	0.6027	1.065
Undec-enedioylcarnitine (C11:1-DC)	1.273	0.2944	0.6269	1.476
Styrene	1.174	0.3011	0.6320	0.772
Prolyl-Tyrosine	1.235	0.3038	0.6320	0.522
Creatinine	1.089	0.3244	0.6537	0.594
Hexenoylcarnitine (C6:1)	1.163	0.3276	0.6537	1.753
Glutamine	1.071	0.3316	0.6537	0.630
Dodeca-trienoylcarnitine (C12:3)	1.202	0.3328	0.6537	0.990
Decatrienoylcarnitine (C10:3)	1.185	0.3377	0.6537	0.620
Tryptophan	1.129	0.3452	0.6537	0.802
Pipecolic acid	-1.140	0.3462	0.6537	0.336
Arginylasparagine	1.173	0.3480	0.6537	0.798
Sphinganine	1.044	0.3495	0.6537	0.898
Creatine	-1.114	0.3503	0.6537	1.203
Aspartyl-Serine	1.181	0.3673	0.6784	0.537

Metabolites with VIP>1	Fold Change[1]	*p*-value	FDR *q*-value at 15%	VIP Score
Hexenoylcarnitine (C6:1)	1.331	0.3735	0.6829	0.460
MethylHexadecanoylcarnitine (C16:0 M)	1.074	0.3953	0.7102	0.588
Oxoproline	1.067	0.3963	0.7102	0.527
Serylcysteine	1.065	0.4223	0.7494	0.464
Hydroxyheptanoylcarnitine (C7:0-OH)	1.197	0.4287	0.7533	0.887
Arginylglutamine	1.141	0.4376	0.7605	0.811
Lactoylphenylalanine	1.165	0.4448	0.7605	0.712
Leucylalanine	1.211	0.4454	0.7605	0.551
Ribose	1.079	0.4718	0.7981	0.730
Serotonin	1.209	0.4789	0.8008	0.523
Phenol	1.079	0.4840	0.8008	0.771
Valerylglycine	1.171	0.4878	0.8008	0.391
Hydroxybutyrylcarnitine (C4:0-OH)	1.223	0.4911	0.8008	0.510
Serylalanine	1.118	0.5038	0.8142	1.096
Octanedioylcarnitine (C8:0-DC)	1.194	0.5094	0.8159	0.875
Leucylproline	1.105	0.5184	0.8178	0.924
Choline	1.061	0.5196	0.8178	0.500
Dec-enedioylcarnitine (C10:1-DC)	1.190	0.5288	0.8251	0.888
Prolylproline	-1.110	0.5697	0.8772	0.582
Salicylic acid	1.110	0.5762	0.8772	0.834
Deca-dienedioylcarnitine (C10:2-DC)	1.091	0.5792	0.8772	0.713
Arginylmethionine	-1.133	0.5816	0.8772	1.231
Tetradeca-trienoylcarnitine (C14:3)	1.102	0.6069	0.9078	0.910
Glutamylvaline	1.117	0.6120	0.9080	0.918
Non-enoylcarnitine (C9:1)	1.059	0.6219	0.9136	1.185

Metabolites with VIP>1	Fold Change[1]	*p*-value	FDR *q*-value at 15%	VIP Score
N-Acetyl-Leu	-1.054	0.6298	0.9136	1.342
Hexadeca-dienedioylcarnitine (C16:2-DC)	1.119	0.6326	0.9136	0.839
Phenylpyruvic acid	1.054	0.6455	0.9136	0.745
Proline	1.039	0.6547	0.9136	0.560
Tridecanoylcarnitine (C13:0)	-1.198	0.6547	0.9136	1.547
Homocysteine	1.099	0.6603	0.9136	1.148
Phenylacetylglutamine	1.133	0.6605	0.9136	0.642
Dodeca-dienedioylcarnitine (C12:2-DC)	-1.160	0.6612	0.9136	1.212
Kynurenine	1.064	0.6909	0.9390	0.913
4-Aminohippuric acid	1.083	0.6923	0.9390	0.778
Octa-dienoylcarnitine (C8:2)	-1.077	0.6952	0.9390	0.924
Undeca-dienoylcarnitine (C11:2)	1.106	0.7105	0.9504	0.938
Methylheptanoylcarnitine (C7:0 M)	1.053	0.7143	0.9504	0.869
Tetradec-enoylcarnitine (C14:1)	-1.168	0.7196	0.9504	1.119
Methylundecanoylcarnitine (C11:0 M)	1.090	0.7252	0.9504	0.661
Methylnonanoylcarnitine (C9:0 M)	1.082	0.7299	0.9504	0.768
Methionine	1.037	0.7352	0.9505	0.865
Glutaric acid	1.072	0.7512	0.9643	0.754
Palmitoylcarnitine (C16:0)	1.034	0.7640	0.9738	0.739
Hydroxytrideca-dienoylcarnitine (C13:2-OH)	1.097	0.7695	0.9740	1.356
Valerylcarnitine (C5:0)	1.044	0.7892	0.9826	0.871
Hydroxypropionylcarnitine (C3:0-OH)	-1.093	0.7900	0.9826	1.028
Methyltetradecanoylcarnitine (C14:0 M)	1.027	0.7973	0.9826	1.646
Threonylserine	1.061	0.8050	0.9826	0.748

Metabolites with VIP>1	Fold Change[1]	*p*-value	FDR *q*-value at 15%	VIP Score
Valylleucine	1.070	0.8064	0.9826	0.460
Dodeca-trienedioylcarnitine (C12:3-DC)	-1.080	0.8264	0.9826	1.098
Butyrylcarnitine (4:0)	-1.037	0.8321	0.9826	1.076
Methyltridecanoylcarnitine (C13:0 M)	1.028	0.8376	0.9826	1.262
Threonine	1.013	0.8543	0.9826	0.590
Hydroxyhexadecanoylcarnitine (C16:0-OH)	1.040	0.8563	0.9826	1.272
Tridec-enedioylcarnitine (C13:1-DC)	1.039	0.8595	0.9826	0.701
Hydroxypentadecanoylcarnitine (C15:0-OH)	-1.057	0.8615	0.9826	0.719
Glutaconic acid	1.025	0.8640	0.9826	1.288
Phenylalanylmethionine	-1.061	0.8649	0.9826	1.216
Tetradeca-dienedioylcarnitine (C14:2-DC)	1.045	0.8692	0.9826	0.839
Glutamylthreonine	1.039	0.8725	0.9826	0.879
Phenylalanylglycine	1.024	0.8929	0.9826	1.210
Hydroxydodeca-dienoylcarnitine (C12:2-OH)	1.027	0.8961	0.9826	0.815
Glycerophosphocholine	1.014	0.8971	0.9826	0.455
Succinylcarnitine (C4:0-DC)	1.024	0.8974	0.9826	0.938
Caprylic acid	1.024	0.8994	0.9826	0.708
Glutarylcarnitine (C5:0-DC)	1.026	0.9012	0.9826	0.759
Nona-dienedioylcarnitine (C9:2-DC)	1.021	0.9062	0.9826	0.880
Acetylcholine	-1.010	0.9143	0.9826	0.935
Arginyllysine	1.026	0.9174	0.9826	0.764
Biotin	1.022	0.9223	0.9826	1.527
Prolylglycine	1.018	0.9229	0.9826	0.762

**Metabolites with VIP>1**	**Fold Change[1]**	***p*-value**	**FDR *q*-value at 15%**	**VIP Score**
Serylglycine	1.012	0.9351	0.9891	0.689
Glutamylproline	1.014	0.9463	0.9891	1.060
Alanyltyrosine	1.018	0.9498	0.9891	0.971
Pyridoxamine	-1.012	0.9513	0.9891	0.866
Hepta-dienoylcarnitine (C7:2)	-1.007	0.9657	0.9891	0.668
Arginine	1.003	0.9722	0.9891	0.472
Tetradeca-trienedioylcarnitine (C14:3-DC)	-1.003	0.9751	0.9891	0.943
Tetradec-enedioylcarnitine (C14:1-DC)	-1.007	0.9755	0.9891	0.793
Lysine	1.003	0.9818	0.9891	1.070
Histidine	1.003	0.9836	0.9891	0.765
Octadec-enedioylcarnitine (C18:1-DC)	1.002	0.9956	0.9956	0.744

[1]Fold change = Mean Term signal with respect to mean Preterm Signal
